# Diversity of two forms of DNA methylation in the brain

**DOI:** 10.3389/fgene.2014.00046

**Published:** 2014-03-10

**Authors:** Yuanyuan Chen, Nur P. Damayanti, Joseph Irudayaraj, Kenneth Dunn, Feng C. Zhou

**Affiliations:** ^1^Department of Anatomy and Cell Biology, Indiana University School of MedicineIndianapolis, IN, USA; ^2^Agricultural and Biological Engineering, Bindley Bioscience Center, Purdue UniversityWest Lafayette, IN, USA; ^3^Division of Nephology, Department of Medicine, Indiana University School of MedicineIndianapolis, IN, USA; ^4^Stark Neuroscience Research Institute, Indiana University School of MedicineIndianapolis, IN, USA

**Keywords:** epigenetics, 5-methylcytosine, 5-hydroxymethylcytosine, chromatin remodeling, histone code, confocal microscopy, FLIM-FRET

## Abstract

DNA methylation 5-methylcytosine (5mC) predicts a compacting chromatin inaccessible to transcription. The discovery of 5-hydroxymethylcytosine (5hmC), which is derived from 5mC, adds a new dimension to the mechanism and role of DNA methylation in epigenetics. Genomic evidence indicates that the 5hmC is located in the alternate regions to 5mC. However, the nature of 5hmC, as compared with classical 5mC remains unclear. Observing the mouse brain through embryonic development to the adult, first, we found that 5hmC is not merely an intermediate metabolite of demethylation, but is long lasting, chromatically distinct, and dynamically changing during neurodevelopment. Second, we found that 5hmC distinctly differs from 5mC in its chromatin affiliation during neural stem cell (NSC) development. Thirdly, we found both 5mC and 5hmC to be uniquely polarized and dynamic through the NSC development. 5mC was found to progressively polarize with MBD1 and MeCP2, and recruits H3K9me3 and H3K27me3; while 5hmC progressively co-localizes with MBD3 and recruits H3K4me2. Critical differential binding of 5mC with MBD1, and 5hmC with MBD3 was validated by Resonance Energy Transfer technique FLIM-FRET. This transition and polarization coincides with neuroprogenitor differentiation. Finally, at the time of synaptogenesis, 5mC gradually accumulates in the heterochromatin while 5hmC accumulates in the euchromatin, which is consistent with the co-localization of 5hmC with *Pol*II, which mediates RNA transcription. Our data indicate that 5mC and 5hmC are diverse in their functional interactions with chromatin. This diversity is likely to contribute to the versatile epigenetic control of transcription mediating brain development and functional maintenance of adult brain.

## Introduction

Since its discovery as the second form of DNA methylation in vertebrate tissue, 5-hydroxymethylcytosine (5hmC), which is most abundant in the brain (Globisch et al., 2010), has been in the eye of the epigenetic storm (Kriaucionis and Heintz, 2009; Tahiliani et al., 2009; Guo et al., 2011; Ito et al., 2011). The discovery of 5hmC opens a new route for established DNA methylation to regulate gene expression. 5hmC, the oxidized form of 5mC by the ten-eleven translocation 1, 2, or 3 (TET1,2,3), can be demethylated, also by TET1,2,3, into 5-formylcytosine (5fC) and further into 5-carboxylcytosine (5caC) (Wolffe et al., 1999; Wu and Zhang, 2010; Bhutani et al., 2011). The TET enzyme and the generation of 5hmC have been found to be involved in programming and maintenance of the pluripotency of embryonic stem (ES) cells (Ito et al., 2010) and totipotency of zygotes (Wossidlo et al., 2011). Current research on 5hmC has primarily focused on its biochemical characterization *in vitro* and, primarily on its involvement in stem cells and germ lines. Roles of 5hmC beyond totipotent cells have been highly anticipated. We first showed that 5mC and 5hmC form a collaborative program leading the neuronal differentiation during neural tube development (Zhou et al., 2011; Zhou, 2012), that prevention of the program retards the differentiation, and the program also occurs in developing as well as adult neuronogenesis in the hippocampus (Chen et al., 2013). Recent finding on genomic reorganization of DNA methylation point to a potential role in synaptogenesis (Lister et al., 2013). To date, the individual function of the 5mC and 5hmC is not clear and is paramount to the understanding of transcription in general, and brain development and function in many ways. While 5mC's association with suppression of transcription is well accepted, the role of 5hmC is unclear (Bhutani et al., 2011). Recent genomic distribution analyses showed that 5hmC and 5mC are distributed in proximity to different categories of histone codes in the embryonic stem (ES) cells (Pastor et al., 2011; Stroud et al., 2011; Williams et al., 2011; Xu et al., 2011), which implicates the potential deviation of their action in ES cells.

Here, we attempt to elucidate the epigenetic role of 5hmC during brain development throughout adult brain. First, we examined 5hmC along with 5mC from early gestation (E8) and throughout the life span of the brain of mice. Second, DNA methylation binding domain (MBD) proteins such as MBD1 and 3 were studied here in conjunction with 5mC and 5hmC. Third, we characterized the relationship with histone codes and chromatin. Since DNA methylation is tissue specific, the dynamic changes between 5hmC and 5mC and their associated chromatin markers including select histone codes and MBDs, were evaluated immunocytochemically. Since the physical proximity of the methylcytosine to MBD is difficult to ascertain by immunocytochemistry, the differential binding of 5mC/5hmC to their MBD partner is further confirmed with high-fidelity Fluorescence Lifetime Imaging based Forster Resonance Energy Transfer (FLIM-FRET).

We report here how 5mC and 5hmC achieve functional diversity through differential temporal appearance, chromatin distribution, and binding partner selection in the brain.

## Materials and methods

### Animals

All mice were used in accordance with National Institute of Health and Indiana University Animal Care and Use (IACUC) guidelines and internationally recognized guidelines. The protocol was approved by the Laboratory Animal Resource Center (LARC) animal ethics committee of Indiana University (protocol ID: 10428). All efforts were made to assure minimal pain and discomfort. C57BL/6 (B6) mice (average 20 grams, 12–14 week old, Harlan, Inc., Indianapolis, IN) were used in this study. Mice were acclimated for at least 1 week before mating. For embryo study, two females were bred with one male for a 2-h period (10:00am–12:00noon). The presence of a vaginal plug at the end of the 2-h mating was considered as indicative of conceptus and that hour was designated as embryonic day (E) 0. On E10 and 17 (each *n* = 8–10), four of the dams were deep anaesthetized with CO_2_ and embryos were surgically removed from the embryonic sack and fixed in 20 ml of 4% ice-cold paraformaldehyde (PFA). Dams were then euthanized with cervical dislocation. The rest of the dams gave birth, which was designated postnatal day (P) 0. On P7, P21, and P45 (*n* = 5–10 each), mice were anaesthetized with CO_2_, and then perfused transcardially with 0.9% saline (100 ml) and 4% formaldehyde in phosphate buffer (0.2 M, pH 7.4). In addition, a group of 3 about 1 year old mice were also used for analysis. The brains were then removed, and post-fixed for at least 24 h at 4°C.

### Immunohistochemistry

**Figure A1:**
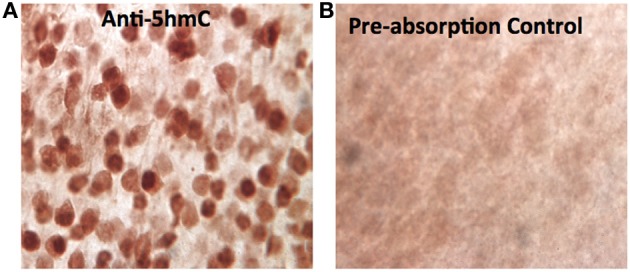
**The specificity of the anti-5hmC antibody**. The specificity of the crucial anti-5hmC antibody against 5hmC was characterized by pre-absorption of anti-5hmC antibody (1:1000, rabbit polyclonal IgG, Active Motif) with 5hmC synthesized DNA (Methylated DNA Standard Kit, #55008, Active Motif, 50 ng/μ l, at a working dilution of 1:20 molar ratio) overnight at 4°C. Before absorption, the anti-5hmC immunostaining in the cortex shows distinct 5hmC staining pattern **(A)** as described in the result, while the staining pattern was abolished or drastically reduced after 5hmC-preabsorption **(B)**.

The embryos and postnatal brain of various ages (E10, E17, P7, P21, P45, and 1 year old) were embedded in gelatin block and fixed with 4% PFA for 48 h and sectioned in 40-μ m-thick coronal sections on a free-floating vibratome (Leica Microsystems; Buffalo Grove, IL) for immunocytochemical procedures. The sections were then cleared of endogenous peroxidases using 10% H_2_O_2_ in phosphate buffered saline (PBS) for 10 min, and permeabilized with 1% TritonX-100 in PBS for 30 min. DNA was denatured with 2N HCL for 30 min, and then neutralized in Tris-HCl (PH 7.4) for 10 min. Sections were blocked using a goat kit containing 1.5% normal goat serum, 0.1% Triton X-100 in PBS for 1 h, then incubated with primary antibodies. Antibodies used were against DNA methylation marks 5mC (1:2000, mouse monoclonal; Eurogenetec, Fremont, CA), 5hmC (1:3000, rabbit monoclonal; Active Motif, Carlsbad, CA; The specificity of the crucial anti-5hmC antibody is characterized by pre-absorption of anti-5hmC antibody with 5hmC; See Figure A1); DNA methylation binding proteins MeCP2 (methyl CpG binding protein 2, 1:1000, rabbit monoclonal; Cell Signaling, Danvers, MA), MBD1(methyl CpG binding protein 1, 1:500, Millipore, Billerica, MA), MBD3 (methyl CpG binding protein 3, 1:1000, Santa Cruz Biotechnology, Dallas, Texas); DNA methylation enzyme Tet1(ten-eleven translocation 1, a 5hmC hydroxylation enzyme; 1:500, rabbit polyclonal; Millipore, Billerica, MA); 5mC metabolites 5-carboxylcytosine (5caC, 1:1000, Active Motif, Carlsbad, CA) and 5-formylcytosine (5fC, 1:1000, Active Motif, Carlsbad, CA); histone marks, trimethyl-H3K4 (H3K4me3, rabbit monoclonal, 1:1000, Millipore, Billerica, MA), trimethyl-H3K9 (H3K9me3, 1:500, Millipore, Billerica, MA), trimethyl-H3K27 (H3K27me3, 1:500, Millipore, Billerica, MA); differentiation markers MAP2 (goat, 1:400, Santa Cruz Biotechnology, Dallas, Texas), anti-nestin (neuroepithelial marker, mouse, 1:500, BD Biosciences, San Jose, CA), and anti-Crabp 1 (cellular retinoic acid binding protein 1, received from Dr. Wei, University of Missouri); and anti-serine-5 phosphorylated RNA polymerase II (*Pol*II) antibodies (Rabbit, 1:1000; Abcam, Cambridge, MA). Primary antibody was diluted in PBS containing 0.1% Triton X-100 and 1.5% normal goat-serum. The next day the sections were washed three times in PBS, and then incubated with the corresponding secondary antibodies conjugated with biotin (Jackson ImmunoResearch, West Grove, PA) for single staining or fluorescent dyes (Invitrogen) and for double staining. For single staining, the sections were further incubated in Streptavidin-AP (1:500, Jackson ImmunoResearch, West Grove, PA) for 90 min and visualized with a brown-colored label, 0.05% 3′-3′-diaminobenzidine (DAB), and 0.003% hydrogen peroxide. The sections were then counterstained with methyl green, and dehydrated. For double staining, a second set of primary antibodies were incubated similarly to the first set followed by secondary antibodies with fluorescent dye (Alexa 488, 546, or 633) for 1.5 h at room temperature. Slices were covered with anti-fade mounting solution with 4′,6′-diamidino-2-phenylindole (DAPI) (Invitrogen). Double-immunocytochemical staining was performed between 5mC and 5hmC, and the 5mC with MBD1, MeCP2, H3K9me3, H3K4me2, or serine-5 phosphorylated *Pol*II; and the 5hmC with MBD3, H3K9me3, H3K4me2, or serine-5 phosphorylated *Pol*II.

### Image acquisition

Confocal fluorescence images were obtained by an Olympus FV1000-MPE Confocal Microscope (Olympus America Inc., Center Valley, PA) mounted on an Olympus IX81 inverted microscope stand with a 60x water-immersed objective lens. Sequential excitation at 488 nm and 559 nm was provided by argon and diode lasers, respectively. Emissions were collected by spectral detectors in channels one and two with user-specified min and max wavelengths. A third channel, collecting fluorescence excited at 405 nm was used for the detection of DAPI. Z-stack images were collected over a thickness of 4.5 μm with 0.3 μm step intervals. Laser intensities and gain values were adjusted to prevent saturation and to reduce the background noise. The same setting was used over the same pair of staining at different brain locations and ages. After sequential excitation, green, and red fluorescence images of the same cell were saved and analyzed by Olympus Fluoview FV10-ASW software (Olympus Corporation 2003–2008).

### Co-localization analysis

For visualization of co-localization of confocal images, different channels of images were overlaid at the same Z-plane. An overlay of green and red would give rise to yellow hotspots where the two molecules of interest were present in the same pixels. Quantification of co-localization was performed with intensity correlation coefficient-based method using an ImageJ plugin JACoP (http://rsb.info.nih.gov/ij/plugins/track/jacop.html). Methods were adapted from (Bolte and Cordelieres, 2006). Pearson's Coefficients (r) were collected for the area of interest in nuclei. The value of r could range from 1 to −1, with 1 denoting complete positive correlation, −1 for negative correlation, and 0 for no correlation. The value of M could vary from 0 to 1, with 0 corresponding to non-overlapping images and 1 reflecting 100% co-localization. Each individual cell was outlined by ImageJ selection tool and measured at the same threshold settings. Only the nuclei with ±10% of the full diameter at section were chosen for the analysis. For each pair of staining, ~20 cells per staining were measured, respectively, from 3 animals per age, and presented as Mean ± SEM. Statistical analyses legitimately evaluating colocalization adopted from McDonald and Dunn (2013) were performed with ANOVA for groups or Student's *T*-test for pair comparisons.

### Fluorescence lifetime imaging based forster resonance energy transfer (FLIM-FRET) analysis

The Immunocytochemistry procedure was done similar to the one described above. In brief, formalin fixed brain tissue section was used. The sections were washed in PBS 3 × 5 min, then permeabilized using Triton-X 1% in PBS for 30 min on a shaker. The first antibody (5mC or 5hmC) was labeled with Alexafluor 488 conjugated secondary antibody at 1:500 dilution in nomal goat serum, on a shaker for 90 min, washed for 3 (3 × 5 min; 3 × 15 min; 4 × 30 min) h. Tissue was then imaged and fluorescence lifetime was measured at the cortex and hypocampus area. The same tissue section was washed for 30 min on a shaker, and then incubated with a pair of primary antibody (MBD1, MBD3, or MeCP2) for 18 h, and labeled with Alexa fluor 546 conjugated secondary antibody at 1:500 dilution for 90 min, and mounted in the imaging chamber for imaging. For each demethylation (5hmc, 5mc)/protein (MBD1, MBD3) interaction 50 cells are used from ~8 cells/setion of hippocampal or cortical region of 4 P7 brains. Total, 400 cells were used for FLIM-FRET analysis.

FLIM-FRET experiment was performed by measuring the fluorescence lifetime of donor in the absence and the presence of acceptor using *Microtime 200* (Picoquant, GmbH, Berlin Germany). Fluorescence lifetime is defined as the time in which fluorescence intensity of a fluorophore is decreased to 1/e of its initial intensity. The source of excitation is a picosecond pulsing diode laser at 467 nm excitation wavelength for the donor (Alexa fluor 488) and 530 nm for acceptor (Alexa fluor 546). The laser repetition rate was 40 mHz, and the laser power was ~3 mW. The laser was focused on the sample using an apochromatic 60x water immersion objective with1.2 NA. Off-focus fluorescence was rejected with a 50 μm pinhole for efficient collection of the emission. Emitted fluorescence was collected using the same objective and separated from excitation beam using dichroic mirror. Band pass filter 480–520 nm for Alexa fluor 488, and 570–645 nm was used to ensure that the collected photons came from the donor fluorophore only. Fluorescence emission was collected using two single photon avalanche photodiodes using (SPAD, SPCM-AQR-14, Perkin-Elmer). Each photon was tagged with a time stamp that identifies its arrival time in the detector after the laser pulse, using time correlated single photon counting (TCSPC) in the Time Tagged Time Resolved Single Photon Mode (TTTR) and the FLIM image and the decay curve were obtained. Details of instrumentation can be found in (Varghese et al., 2008; Chen et al., 2012).

Fluorescence lifetime was obtained from the multiexponential reconvolution fitting with the appropriate instrument response function (IRF), following (Lakowicz, 2010; Chen et al., 2011):

$$I(t)=\sum_{i\;=\;1}^{n}\alpha_{1}\exp\;(-\frac{t}{t_{i}})$$

Time bin was applied to remove the background and threshold was set for 500 photon per pixel. A minimum of 25 different cells from 4 to 5 separate experiments were fitted for efficiency calculations.

FRET efficiency (Vidi et al., 2008) was calculated by:

$$\text{Efficiency}=1-\frac{\tau a}{\tau b}$$

where: τa = Fluorescence lifetime of donor with the absence of acceptor τa = Fluorescence lifetime of donor with the presence of acceptor Efficiency above 5% is considered as significant and vice versa (Ruttinger et al., 2006).

**Figure A2:**
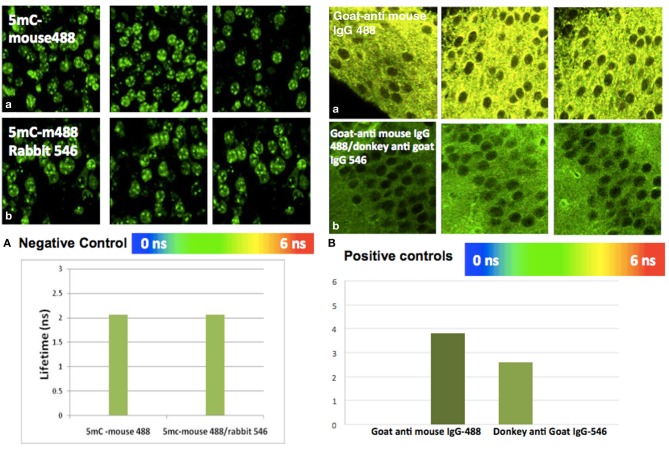
**Positive and negative control study by FLIM-FRET**. **(A)** Negative control: There is no interaction between mouse IgG Alexa488 and anti Rabbit IgG Alexa546. **(a)** Average lifetime anti-5mC mouse IgG Alexa488 was 2.128 ns. **(b)** Average lifetime of anti-5mC mouse IgG Alexa488 and mouse anti rabbit-ant IgG Alexa546 was 2.120 ns. There is no significant change (FRET efficiency >0.05 and at *P* > 0.05) between mean lifetime of donor and acceptor in hippocampus. **(B)** Positive controls: There is interaction between goat/anti-mouse IgG Alexa488 and anti-goat IgG. **(a)** Average lifetime of goat-anti mouse IgG Alexa488 was 3.8 ns, while **(b)** the average lifetime of goat anti-mouse IgG Alexa488 and anti-goat IgG Alexa546 was 2.6 ns. There is significant change (FRET efficiency >0.05 and at *P* < 0.05) between mean lifetime of lifetime donor and acceptor in the hippocampus.

Both the positive and negative controls were also performed by FLIM- FRET to validate our findings. For positive control, we demonstrated that the goat anti-mouse IgG Alexa 488 and donkey anti-goat Alexa 546 IgG (depicting co-localization of the two fluorophores, acceptor and donor,) were within the FRET distance denoting co-localization of the two marks. For negative control, the primary mouse anti-5mC and the secondary anti-mouse Alexa488 donor (blue channel) does not show any binding proximity to the anti-rabbit Alexa 633 or anti-rabbit Alexa 546 (Acceptor red channel). (See Figure A2).

## Results

### Ontogeny of DNA methylation of the nervous system

**Figure 1:**
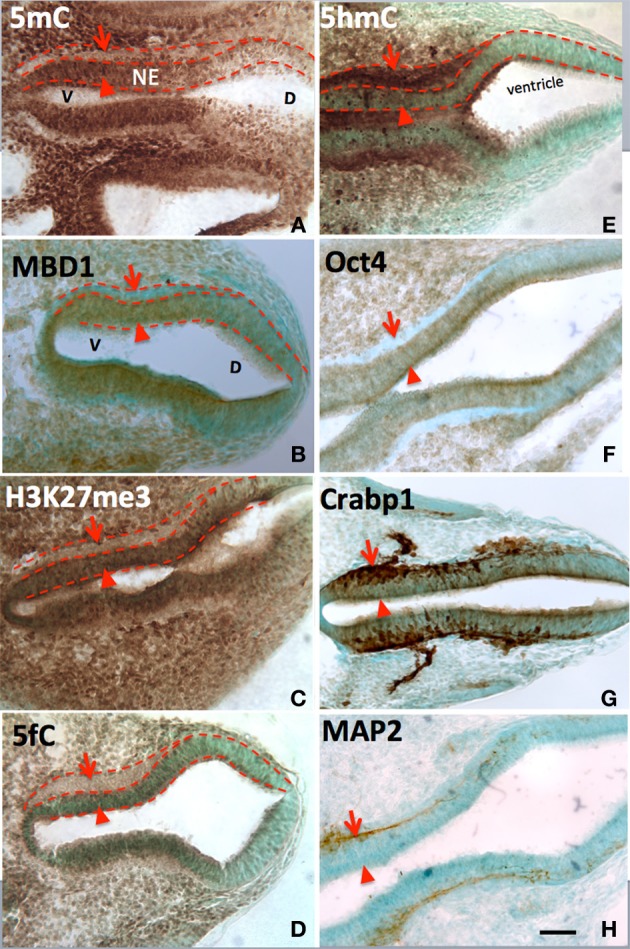
**The differential role of 5mC vs. 5hmC in the DNA methylation program is inferred by their association with phenotypic marks during the making of neural tube**. At early neurulation (E10), a sequential escalation of 5mC and 5hmC occurred, where 5mC appeared first at neuroepithelial (**A**, NE) layer (arrowheads) to prepare the differentiation, and faded as the NE cells differentiated and migrate away from ventricle (**A**, arrows). At this time, the distribution of the DNA methylation binding protein, MBD1, has exactly the same pattern as that of 5mC **(B)**. Its function is depicted by the suppressive histone mark, H3K27me3, which distributed **(C)** similar to that of 5mC and MBD1. The demethylated metabolite 5fC also matched with 5mC. In contrast, the 5hmC appeared at differentiating neurons (**E**, arrowheads) and continued to increase in differentiated neurons (**E**, arrows). This program also occurred at ventro- (left) dorsal (right) gradation **(A–H)** where 5mC **(A)** preceded 5hmC **(E)**. The role of 5mC and 5hmC on neural tube cell differentiation is further depicted by the differentiation phenotypic markers in a spatially precise manner. The role of the 5mC is demonstrated by the appearance of the Oct4 in the NE layer (**F**, arrowhead) and the loss of Oct4 in differentiated neurons (**F**, arrow). The role of 5hmC is noted by the stage-wise neuronal marker Crabp1 **(G)** and MAP2 **(H)** distributed in a spatially correlated manner. V, ventral; D, dorsal. Scale bar: All = 100 μm. Color scheme: Immunoreactive DAB, brown; counterstaining, methyl green.

At every point of neural differentiation in the neuroepithelial cells throughout the neural tube and brain development, the immunostained 5mC and 5hmC appeared or surged in tandem–first the 5mC followed by 5hmC hours to days later. At embryonic day (E) 8-E10, 5hmC and 5mC showed a distinct pattern in the ventricle subpopulation of the neural tube. 5mC increased in a spatiotemporal gradation, axially from the hindbrain to rostral and caudal neural tube, and at each level from ventral to dorsal aspects. This 5mC distribution gradient was found to correlate spatiotemporally with the differentiation gradient in the neural tube as reported previously (Zhou et al., 2011). In this report we reveal that, it is the sharp increase of 5hmC, hours to days after the increment of 5mC that marks the beginning of neuroepithelial cell differentiation. Stages of differentiation were marked by the sequential appearance of nestin, cRABP, and MAP2 staining (Figure 1). Immunocytochemical staining of 5hmC coincided with that of Tet1/2 (Figure 1). Throughout all the regions of the differentiating neural tube, the surge of 5hmC staining can be related to a particular stage of differentiation. Both 5mC and 5hmC clearly increased as differentiating neurons migrated toward their targets.

**Figure 2:**
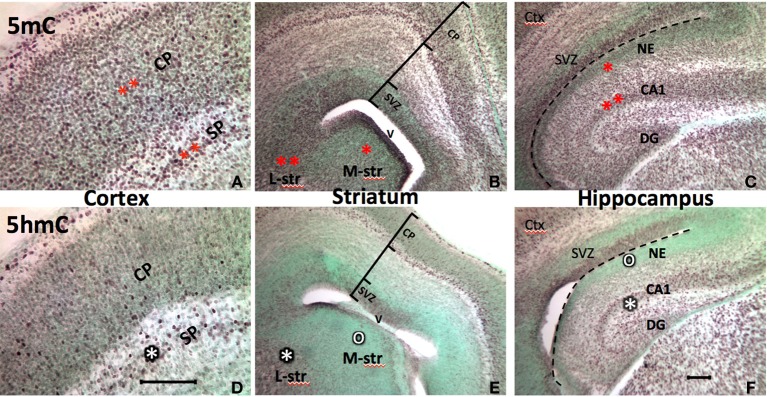
**Progression of the 5mC and 5hmC commonly parallels with the neuronal differentiation throughout the developing brain**. Also, the sequential escalation of 5mC precedes 5hmC at the stage of neural differentiation in the cortex (**A** vs. **D**), striatum (**B** vs. **E**), and hippocampus (**C** vs. **F**) (The degree of methylation is shown by the number of stars, where lack of methylation is denoted by “o”). At the stage of early brain formation at E17, the 5mC appeared in the neuroepithelial cells preempting for differentiation (e.g., medial striatum and hippocampal neuroepithelium; **B**,**C**, one red star area), and continue in the differentiating neurons in cortex **(A)**, lateral striatum (L-str, **B**), and CA regions (**B**, two red stars). The 5hmC arrives in the cells initiated for differentiation, and the level of 5hmC parallel with the stage of differentiation, e.g., subplate>cortical plate **(D)**, lateral striatum (L-str)> medial striatum (M-str) (**E**, white star), CA1>neuroepithelium (**F**, white star). Counterstaining, methyl-green (green color). SVZ, subventricular zone; CP, cortical plate; SP, subplate; NE, neuroepithelium; V, ventricle; DG, dentate gyrus. Scale bar: **A** and **D** = 100 μm; **B,C,E,F** = 100 μm.

The epigenetic pattern in the developing brain is very similar to that of the neural tube. At early postnatal stage (P7), 5mC and 5hmC continue to evolve in the differentiating neurons in the brain where neuroepithelial cells are undergoing differentiation, while less 5mC-immunostaining (im) and no 5hmC-im are noted in the undifferentiated neuroprogenitor cells in the forebrain. For example, an intense 5mC-im and moderate 5hmC-im appeared in differentiated neurons in the lateral striatum, but a low concentration of the 5mC-im and no 5hmC-im appeared in the immature primordial cells in medial striatum at P7 (Figure 2). The timing and partnership of 5mC and 5hmC are well demonstrated spatiotemporally in layer development of archicortex (e.g., hippocampus) (Chen et al., 2013) and the neocortex (e.g., frontal cortex) (Figure 2). In more mature brain, e.g., at P45, higher amounts of 5mC and 5hmC are distributed in all neurons and glial cells, except the neural progenitor cells in the subventricular zone and subgranular layer of the dentate gyrus where, 5mC-im appeared in low level and 5hmC-im is mostly absent or extremely low, recapitulating the prenatal early neurogenesis. Apparent diverse density of the DNA methylation marks are evident in different regions of the mature brain e.g., striatum vs. cortex, and hippocampus (Figure 2) (As a note, the cellular methylation level in the cerebellum is not exceptionally higher than other regions of the brain as previously indicated *in vitro* (Kriaucionis and Heintz, 2009). High levels may be due to the biochemical measurement in the high-density granule cells, or a discrepancy between the *in vitro* and *in vivo* analysis. Within a region, different intensities of 5mC-im and 5hmC-im were also found in different populations of the neurons during the developmental stages. The intensity of 5hmC in the brain cells varied to a greater degree than that of 5mC. Both 5mC and 5hmC were maintained throughout the young adult stage and in the 1-year-old. A reduction of 5hmC and 5mC were found in both neurons (in gray matter) and glial cells (in white matter) in ~1-year-old brain.

### Chromatin dynamics

**Figure 3:**
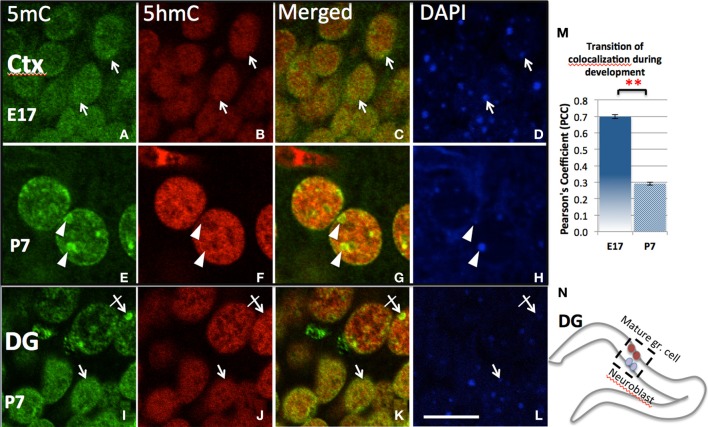
**Differential chromatin remodeling of the 5mC (green) and 5hmC (red) during neuroprogenitor cell differentiation**. In the developing cortex, both 5mC and 5hmC were diffusely distributed in undifferentiated cells and many were overlapped at E17 (**A–C**, **D**:DAPI. also see quantitation by Pearson's Coefficient analysis in **M**), but polarized into different compartments (**E–G**; arrows) as the neuron differentiated at P7. The 5mC (**E**, arrowheads) became associated with DAPI-dense heterochromatic region (**H**, arrowheads), while 5hmC in contrast with DAPI-sparse eurchromatic region (**B,F**, arrowhead). The dissociation is indicated by decrease of Pearson's Coefficient between E17 and P 7 shown in chart m. ^**^P < 0.005. Similar remodeling was found in the two differentiating stage of cells within the P7 dentate gyrus (DG; **I–L**), where undifferentiated or less differentiated (in inner layer, see **N**) and differentiated cells (in outer layer) are layered (see **N**). The 5mC and 5hmC were both homogeneously distributed in younger granule cells (**I–L**, arrows), but 5mC was translocated into heterchromatic DAPI-dense compartments (**I–L**; crossed arrow), while 5hmC was located in euchromatic DAPI-sparse compartment avoiding the heterchromatic DAPI-dense granules (**J,K**, crossed arrow) in the mature granule cells. Scale bar: **(A–L)** = 10 μm. All staining is within nuclei.

Initial observation of the surge of 5mC and 5hmC by confocal microscopy indicated a diffusely distributed pattern in the nucleus (e.g., in ventral neural tube at E10, or in cortical ventricular zone at E17). The degree of co-localization was indicated by Pearson's Coefficient and Manders' Coefficient analysis (see Methods). As the neuroprogenitors progressed to differentiation, 5mC and 5hmC co-localization progressively decreased and were finally found in a characteristic complementary mosaic chromatin compartment in the differentiated state, which peaked at early postnatal days (e.g., P7 in the differentiated cortical, hippocampal CA neurons, and lateral striatum). Thus, the 5mC and 5hmC within differentiated neuronal nuclei are distinctively distributed in different chromatin compartments, and differentially associated with varying chromatin states. The 5mC-im distributed in 4′,6′-diamidino-2-phenylindole (DAPI)-dense aggregates of heterochromatin regions (~0.5–1.5 μm) is completely void of 5hmC-im. The 5hmC-im is solely distributed in fine, DAPI-sparse euchromatic regions (<0.3 μm), which also contain small amount of 5mC (Figure 3). Chromatin remodeling is revealed by the co-localization analysis over a period of time. The mosaic distribution of the 5hmC and 5mC is also demonstrated by their association with DNA methylation binding proteins and histone codes associated with gene transcription states shown below.

### Differential binding partners

**Figure 4:**
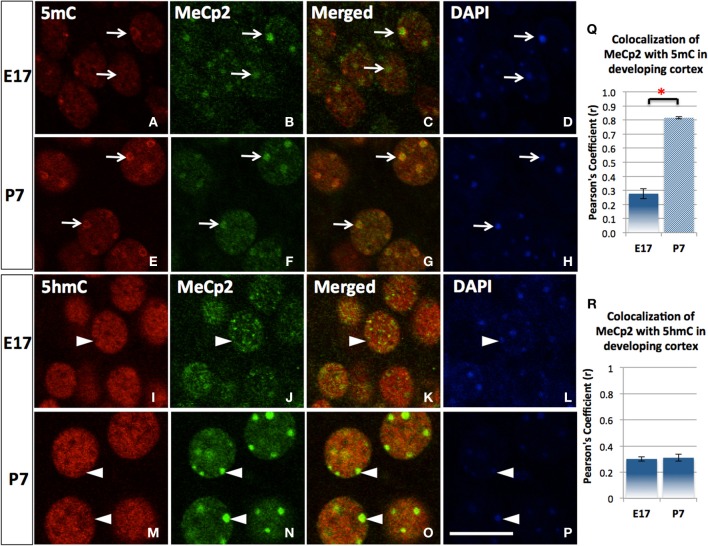
**The transitional association of MeCP2 from 5mC to 5hmC during neuronal maturation**. The association of MeCP2 with 5mC is consolidated as neurons mature shown in the cortex. Nuclear distribution of methyl-binding protein 2 (MeCP2) is homogeneous and mildly associated with 5mC **(A–D)** at E17 but greatly increased at P7 in cortex **(E–H)**. Pearson's Coefficient (r) for co-localization was shown in the chart **(Q)**. In contrast, the homogeneously (euchromatic) distributed MeCP2 colocalized with 5hmC at E17 (**I–L**, arrowhead), but the aggregated (heterochromatic) MeCP2 dissociated with 5hmC (colocalized instead with 5mC as shown in **E–H**) in more mature neurons at P7 (**M–P**, arrowhead). Pearson's coefficient for co-localization was shown in chart **(R)**. Scale bar: All = 10 μm. It is shown that MeCP2 (**D,N**; arrowheads) are mostly distributed at DAPI-dense region with heterchromatin (**D,H**; arrowheads) where 5hmC is usually absent (**I,M**; arrowheads). Scale bar: all = 10 μm. ^*^p < 0.05.

**Figure 5:**
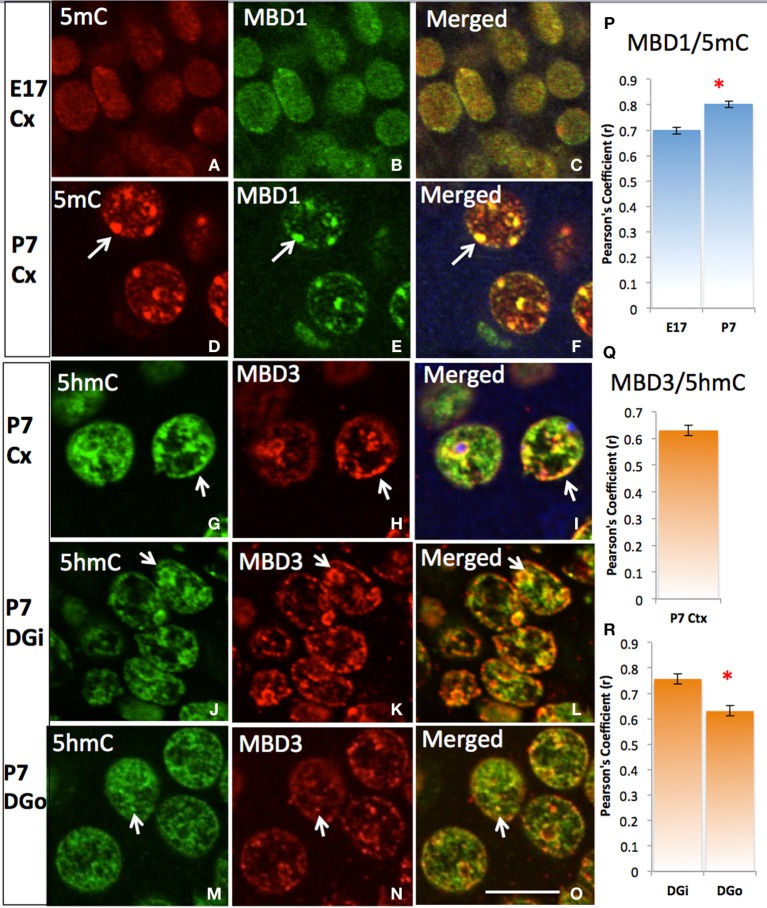
**Differential DNA methylation binding protein partners and their co-translocation during chromatin remodeling over the stage of neuronal maturation**. Two color-fluorescent confocal microscopy shows that the 5mC (red) predominantly co-localized (**A**, red) with MBD1 (**B**, green) initially in the homogeneous small punctate form at nuclei of young E17 neurons at layer IV of cortex (Cx) **(C)**, and increasing their co-localization **(P)** and strikingly co-located in the heterochromatin aggregates when became more differentiated by P7 (**D–F**, arrows). The colocalization of 5hmC (**G**, green) with MBD3 (**H**, red) were seen at E17 (not shown) and P7 (**G–I**, Pearson's Coefficient is shown in **Q**) in the cortex and in the dentate gyrus **(R)**. The 5hmC co-locates with MBD3 to homogeneous euchromatic region (DAPI sparse, now show) from young neurons at inner layer of dentate gyrus (DGi, **J–L**) to older outer layer dentate gyrus (DGo, **M–O**). Scale bar: All = 10 um. The Pearson's Coefficient analyses of colocalization are shown on **(P–R)**. ^*^p < 0.05.

5mC and 5hmC are differentially associated with methyl CpG binding domain (MBD) protein in neurons throughout the brain (e.g., cortices, hippocampus, and cerebellum). By confocal microscopy, double staining revealed a distinct partnership between MBDs and the 5mC and 5hmC. MeCP2 appeared in the neural tube where neurons formed as early as the mouse embryo day 10 (E10) following the appearance of 5mC by approximately a day. The 5mC co-localized with the MeCP2 with remarkably high-fidelity (Figure 4). The co-localization was found in the large DAPI-dense aggregates as well as in the fine DAPI-sparse chromatin particles in the differentiated neurons. In contrast, the 5hmC, which is devoid of DAPI-dense aggregates, does not overlap with aggregated (heterochromatic) MeCP2 in either compartment of the chromatin (Figure 4). Co-localization of 5mC and its binding partners MeCP2 and MBD1 evolved as the brain developed, with co-localization of 5mC and MeCP2 occurring prior to that of 5mC and MBD1. The 5mC and MBD1 which were partially colocalized and homogeneously distributed in DAPI-sparse regions in early development (e.g., at E17), became highly colocalized and translocated to mostly heterochromatic aggregates in the cortex (Figure 5) and other brain regions of similar maturation stage. In contrast, the 5hmC-im is highly co-localized with MBD3 throughout specific brain regions, e.g., in cortex (Figure 5). The 5mC and MBD3 localization in contrast has been low (not shown).

**Figure 6:**
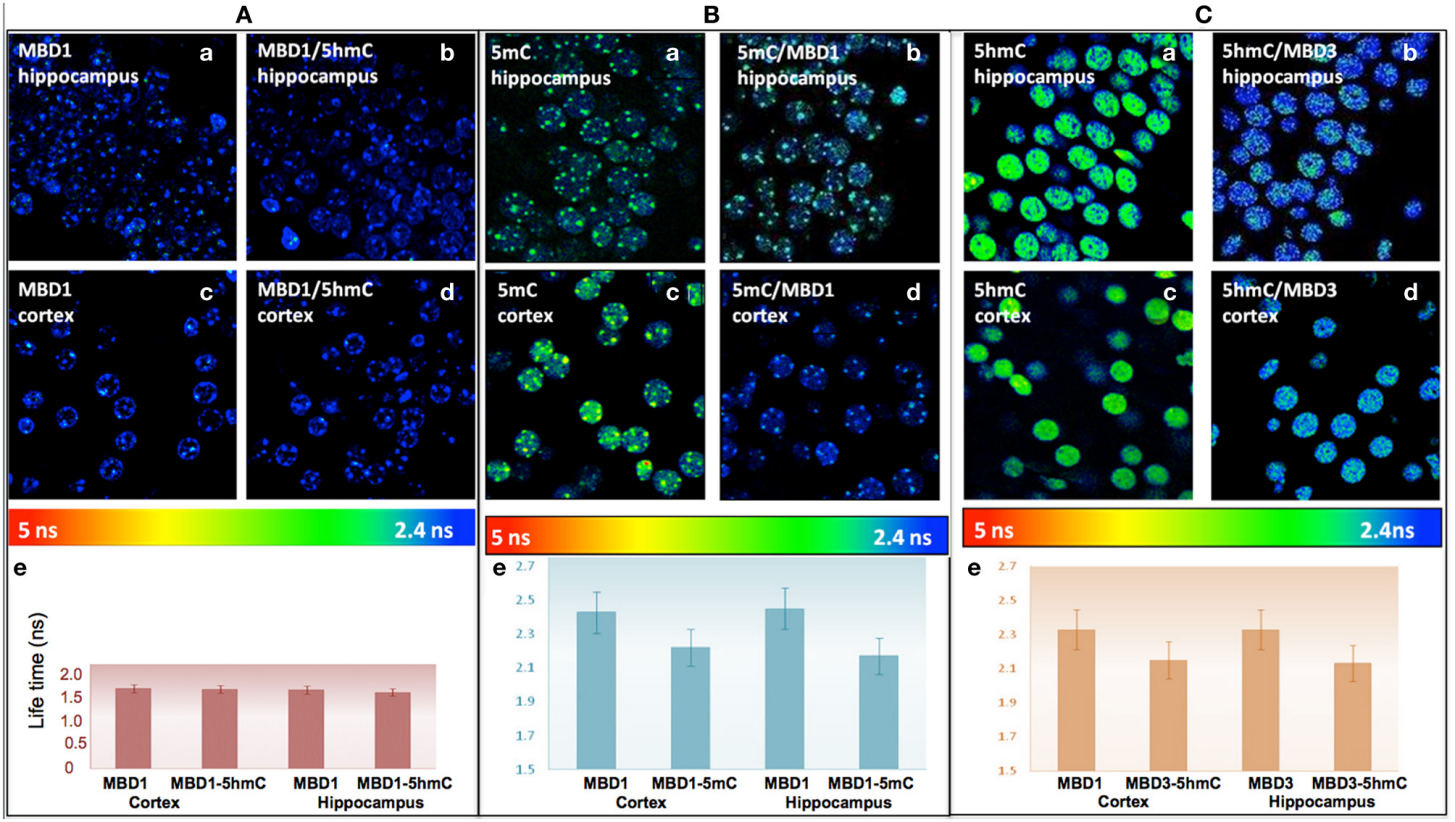
**(A)** There is no significant FRET between MBD1 and 5hmC in the hippocampus **(a)** MBD1 **(b)** MBD1/5hmC, and in cortex **(c)** MBD1 **(d)** MBD1/5hmC. There is no significant change (*P* > 0.05) (FRET efficiency <3%) of the lifetime of donor in the cortex and hippocampus. Average lifetime **(e)** of MBD1 was 1.53 ns, and MBD1/5hmC was 1.51 ns in cortex, and MBD1 was 1.5 ns and MBD1/5hmC was 1.47 ns in the hippocampus. **(B)** There is a significant FRET between 5mC and MBD1 in hippocampus: **(a)** 5mC **(b)** MBD1/5mC, and in cortex **(c)** 5mC **(d)** 5mC/MBD1. There was a significant decrease (*P* < 0.05) (FRET efficiency >5%) in the lifetime of donor in the cortex and hippocampus. Average lifetime **(e)** of 5mC was 2.45 ns and 5mC/MBD1 was 2.17 ns in cortex, 5mC was 2.42 ns and 5mC/MBD1 was 2.22 ns in the hippocampus. **(C)** There is a physical interaction between 5hmC and MBD3 in hippocampus (**a**, 5hmC; **b**, MBD3/5hmC) and in cortex (**c**, 5hmC; **d**, 5hmC/MBD3). There is a significant decrease (*P* < 0.05) (FRET efficiency >5%) of lifetime donor in cortex and hippocampus. Average lifetime **(e)** of 5hmC was 2.45 ns, and 5hmC/MBD3 was 2.17 ns in cortex; and 5hmC was 2.42 ns, and 5mC/MBD3 was 2.22 ns in the hippocampus.

To better enumerate and validate the binding partners, DNA methylation-MBD interaction in real-time, Fluorescence Lifetime Imaging based Forster Resonance Energy Transfer (FLIM–FRET) approach was utilized for precise evaluation of the interactions. We demonstrated that there was low interaction between 5hmC and MBD1 in either hippocampus (FRET efficiency 2.98%) or cortex (FRET efficiency 1.11%) (Figure 6A). In contrast, strong interaction between 5mC and MBD1 (FRET efficiency 8.57–9.65%) (Figure 6B) and between 5hmC and MBD3 (FRET efficiency 7.68–8.40%) in both hippocampus and cortex (Figure 6C) were noted.

### Differential association with specific code of histone and transcription site

**Figure 7:**
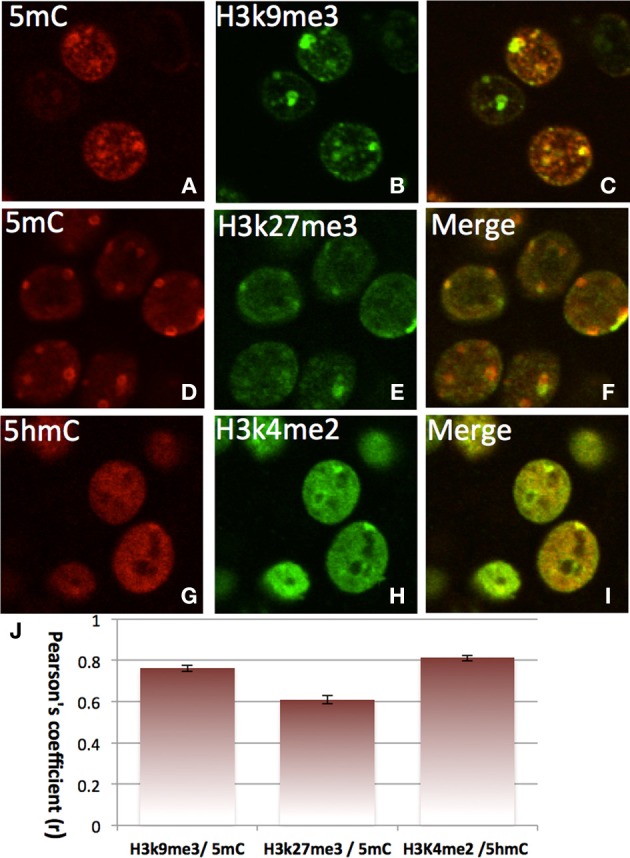
**Differential colocalization of 5mC and 5hmC with histone marks in nuclei of differentiated neurons in P7 cortex indicated by two color-fluorescence confocal microscopy**. The 5mC (**A,D**, red) is closely colocalized with suppressive histone marks H3K9me3 (**B**, green) (merged in **C**), and H3K27me3 **(E**, green) (merged in **F)**. In contrast, the 5hmC **(G**, red**)** is highly co-localized with the activating histone mark, H3K4Me2 **(H**, green**)** as demonstrated in the merged image **(I)**. Pearson's coefficient is shown in chart **J**. Data presented as Mean ± SEM.

The double-staining of DNA methylation with histone codes indicated that 5mC favorably co-localizes with H3K9me3 and with H3K27me3, which are mostly located in the DAPI-dense aggregates (Figure 7). On the contrary, 5hmC does not co-localize with H3K9me3 or H3K27me3. Instead, it co-localizes with H3K4me2 in the DAPI-sparse regions while a lack of co-localization between 5mC with H3K4me2 (Figure 7) is noted. The divergent 5mC and 5hmC co-localization with histone codes is independent of the development stage (e.g., E17, P7, P28) and is observed throughout the brain (the cortices, hippocampus, brainstem, and cerebellum).

**Figure 8:**
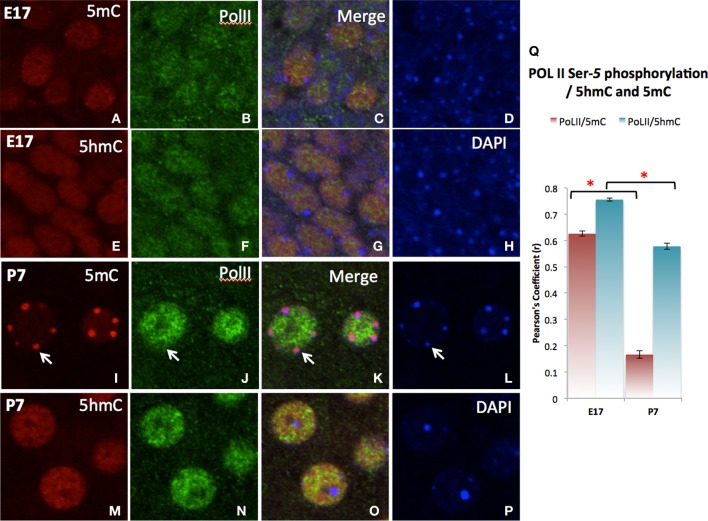
**Differential co-localization of *Pol*II (transcription elongation enzyme) with 5hmC over 5mC in the cortex as demonstrated in confocal microscopy**. At stage of early differentiating (E17), both 5mC **(A–D)** and to a greater extend 5hmC **(E–H)** are associated the *Pol*II. As neurons mature (P7), *Pol*II dissociates with 5mC **(I–L,Q**, arrows**)**, while specifically associates with 5hmC **(M–Q)**. The 5hmC (red) and *Pol*II (green) sites are all in the euchromatic regions exclusive from DAPI stained heterochromatic region (blue, Arrow). Pearson's analysis of co-localization of DNA methylation marks and *Pol* II indicates that the initial co-localization between 5mC and *Pol*II were notedly was diminished at the later stage **(Q)**. This observation summarizes the differential role of 5mC and 5hmC on transcription. ^*^ *p* < 0.05.

The differential role of 5mC and 5hmC is summarized by its association with the *Pol*II transcription enzyme for transcription initiation (Serine-5 phosphorylated *Pol*II) (Cho et al., 2001; Cheng and Sharp, 2003). A strong co-localization of 5hmC with *Pol*II was shown during neuronal differentiation throughout the emerging (E17) stage and slightly decreased in more matured (P7) neurons as demonstrated in the cortex (Figure 8). The co-localization between 5mC and *Pol*II was transient and dissociated as neuronal differentiation progressed at P7 as indicated by the Pearson's coefficient (Figure 8I).

### Stable existence of demethylation intermediates 5hmC, 5fC, and 5caC

**Figure 9:**
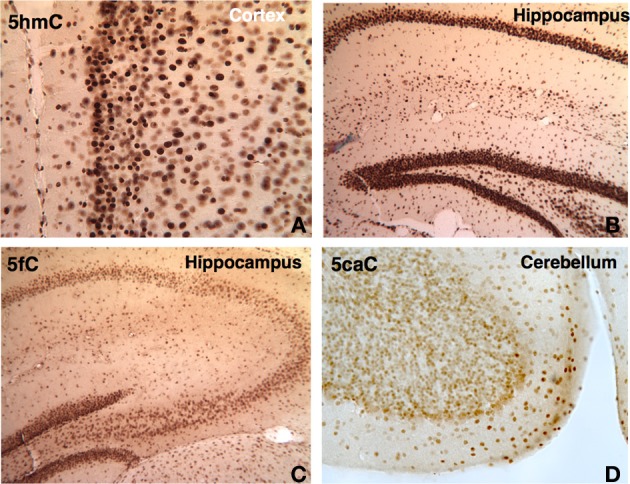
**DNA methylation intermediates were observed to be persistent throughout the life in the brain**. A few examples are shown here. The 5hmC persists in the cortex **(A)** and hippocampus **(B)** in 1 year-old brain. The demethylated cytosine marks also prevailed. The 5fC is shown in the hippocampus at P21 **(C)**, and 5caC **(D)** in cerebellum at P45.

Although a slight reduction in the subpopulation of cells was observed, the 5mC and 5hmC remain throughout, in ~1 year-old brain (e.g., thalamus, basal ganglia) compared to that of young adult. 5hmC's appearance in the prenatal stage shows that it is a prominent feature in neural cells, and persists throughout the life span in the brain as observed in our early time points of E8 and E10, to P7, P21, P45, and 1 year-old (Figure 9). Apparently, 5hmC is not a temporary intermediate as noted from samples obtained from different ages of the brain. We next observed that the demethylation forms, 5fC and 5caC, which exist in smaller concentration compared to that of 5mC and 5hmC, appear during early development (E10, E17) and exist in the nervous system from the prenatal stage throughout adulthood (e.g., P21, P45) (Figure 9). Thus, it is evident that the demethylation intermediate and the demethylated cytosine variants are lasting marks on DNA.

## Discussion

### 5mC and 5hmC transition during development

5mC and 5hmC co-distribute as early as in the zygote stage, where most of the DNA methylation is inherited. Global demethylation eliminated most of the DNA methylation in the early zygotic development during implantation (Kafri et al., 1993; Morgan et al., 2005; Inoue et al., 2011; Iqbal et al., 2011; Salvaing et al., 2012). We found that during neurulation there is a sharp escalation of DNA methylation. The escalation is not random but rather progressed in a pattern closely associated with the state of differentiation of the neuroprogenitor cells. Both 5mC and 5hmC progressed in the newly differentiated neuroepithelial cells as observed from the spatiotemporal patterns in the neural tube and developing brain. However, there are two major differences between the two DNA methylation marks. First, the temporal surge of 5mC and 5hmC is punctual (at very specific stage of neural differentiation) and step-wise (always escalation of 5mC first and followed with 5hmC) throughout developing pre- and postnatal brain (where neural differentiation occurs). The latter is not surprising since it is in agreement with the sharp rise of the TET enzyme, which oxidizes 5mC into 5hmC (Figure 1). However, very interestingly, it is the timing of the surge of 5hmC, but not 5mC, that marks the initiation of neural progenitor cell differentiation. Our experiments show that the escalation of 5mC coincides with the shutdown of proliferation just prior to neural stem cell differentiation; and that the surge of 5hmC, which is involved in transcription, coincides with neural differentiation. The transition from 5mC to 5hmC upon initiation of progenitor cell differentiation and determination of cell fate is ubiquitous throughout the brain regions observed and depending on the stage of neural stem cells instead of overall age of the brain. This pattern is carried into the adult neural stem cells, where 5mC and 5hmC sequentially escalated in newly stem post-mitotic mature neurons, while little-to-low 5hmC is observed in undifferentiated adult progenitor cells near the ventricle and in the hippocampal sub-granular layer (Chen et al., 2013).

### 5hmC is not a transient intermediate

Is 5hmC merely an intermediate state of demethylation, serving no biological function? Recently, instead of being transient and unstable, 5hmC was detected as a relatively stable epigenetic marker in ES or zygotic cells (Inoue and Zhang, 2011; Iqbal et al., 2011). The following evidence lead us to believe that 5hmC is not a transient intermediate of demethylation, although it is not without turnover, as we previously indicated in the ontogeny of DNA methylation (Zhou et al., 2011; Zhou, 2012). Our observations that 5mC and 5hmC co-distribute throughout neurulation and brain development, and that 5hmC exists and persists throughout the life span of neurons strongly argues against the notion that 5hmC is merely an intermediate product. Further, we found the demethylation products 5fC and 5caC, which are also recently being considered for potential functionality (Burgess, 2012; Kellinger et al., 2012), are stably expressed in the developing neurons and throughout the mature brain. Taken together, this evidence support our claim that that 5hmC is a long-standing and dynamic component.

### 5hmC is a league of its own

Is 5hmC a genuinely new breed of DNA methylation? Apparently, 5hmC and 5mC can be discriminated via their binding partners. Our evidence thus far suggests that at least they are read by distinctly different binding proteins—5mC preferentially binds to MeCP2 and MBD1, while 5hmC preferentially binds to MBD3. Recent reports indicate a mix view on the association of 5hmC vs. 5mC to MBD3 (Yildirim et al., 2011; Hashimoto et al., 2012; Spruijt et al., 2013). All these studies were conducted *in vitro* and mostly in ES cells. Given the dynamic nature of DNA methylation, the evolution of the respective binding partner is also likely dynamic, potentially resulting in differences between *in vitro* and *in vivo* interactions. Our observations, obtained throughout brain development demonstrated that there is a dynamic transition of this differential binding coincident with neuronal differentiation through the neural developmental stage (Figure 5). The bona fide differential binding activities of 5mC with MBD1 and 5hmC with MBD3 partners are demonstrated here by the FLIM-FRET analysis for the first time (Figure 6). This observation affirms the previous finding which points to the fact that in differential genomic distribution that 5mC is preferentially localized in the promoter regions of the gene, while 5hmC and MBD3 is preferentially distributed in the downstream transcription starting sites (TSS) in the gene body regions (Stroud et al., 2011; Wu et al., 2011; Yildirim et al., 2011).

Functionally, the genes associated with 5hmC in the gene bodies are actively transcribed in the mouse embryonic stem cells and mouse cerebellum (Guo et al., 2011; Pastor et al., 2011; Wu et al., 2011). The functional diversity of the two forms of DNA methylation is further suggested in the current study by their cellular affiliation first with correspondent histone codes—5mC with the suppressive H3K9me3 and with H3K27me3, mostly in the heterochromatin regions; and 5hmC with H3K4me2 in the euchromatin regions (Figure 7). This methylation transition and histone code affiliation is also demonstrated at the genomic level. 5hmC is enriched in the TSS in the gene body close to regions of H3K4me2, but not near H3K9me3 regions. Further, 5hmCs are also enriched in regions associated with activating the histone codes H3K4me1 and H3K27ac (Stroud et al., 2011). In genes, 5hmC transitions from silent to activated, and was found to affiliate with the activating H3K4me3 and suppressive H3K27me3 in ES cells (Pastor et al., 2011; Williams et al., 2011). Perhaps the strongest evidence that we have observed is the co-localization of 5hmC with *Pol*II in the euchromatin regions throughout neuronal differentiation, and the transient and complete dissociation of 5mC and *Pol*II throughout chromatin in nucleus (Figure 8). Together, these observations indicate that the conversion of 5mC to 5hmC is a dynamic process that may serve as a means for transcriptional transition to regulate ES cells in previous studies (see introduction) and those of the developing nervous system (Zhou et al., 2011; Zhou, 2012; Chen et al., 2013). On the other hand, after 5hmC has settled in mature neurons, significantly long-term high levels of 5hmC, as well as 5fC and 5caC, were observed through adulthood. This indicates that 5hmC is not a transient intermediate toward demethylation, rather it is highly associated with euchromatin and likely maintains the activity of genes for cell specificity.

### Chromatin remodeling

The significance of the escalation of 5mC and 5hmC during the course of neuroepithelial cell differentiation has to do with chromatin remodeling and DNA packaging. We found that the selective DNA methylation and histone marks are progressively distributed differentially to correspond with the DAPI-dense vs. -sparse chromatin compartments. In the developing brain, at perinatal stage (E15-P7), 5mC relocates from DAPI sparse to the DAPI-dense heterochromatin, while 5hmC affiliates with DAPI-sparse euchromatin. The 5mC and 5hmC associated chromatin remodeling peaking at P7 coincides with synaptogenesis. Another event of DNA methylation reconfiguration—where CpG methylation (mCG) is switching to non-CpG methylation (mCH) also coincides with synaptogenesis (Lister et al., 2013). These two events highlight the dynamics of DNA methylation during brain development.

The above distinct differential affiliations provide a major clue suggesting a departure of 5hmC from 5mC and a transition toward transcriptional activation. It infers that the transition from 5mC to 5hmC is pertinent to chromatin remodeling and to DNA packaging in the different compartments for the silenced vs. activated gene cohorts for timely functional or developmental progression during neural fate determination or tissue specification. The differential association of 5mC with suppressive histone codes, and 5hmC with activation histone code, further support chromatin remodeling to organize the transcription transition. An epigenomic-gene expression analysis in the hippocampus and cerebellum (Szulwach et al., 2011) supports this view. Meehan et al. (Nestor et al., 2012) recently further demonstrated that 5hmC is associated with transcribed genes.

## Conclusions

We demonstrated that 5mC dramatically increased in the neuroprogenitor cells during priming for differentiation, and preferentially binds to MBD1 (as indicated by FLIM-FRET), and colocalizes with MeCP2, histone H3K9me3 and H3K27me3, and is finally packed into heterochromatin during and after neural differentiation. 5mC is likely to organize a more selective transcription during differentiation. In contrast, 5hmC surged at the initiation of differentiation of neuroepithelial cells, preferentially bound to MBD3, colocalized with the euchromatin histone H3K4me2, and gradually translocated to the euchromatin, demonstrating a transition to transcription that is needed for differentiation. Thus, 5mC and 5hmC, which divided into different chromatin and differentially associated with distinct functional partners through development and remained in adulthood, likely represent functionally unique DNA methylations in the epigenetic landscape. This added complexity in the epigenome may increase the efficiency with which the complex journey of development is accomplished, and may improve the responsiveness of the system to the environmental inputs (Zhou, 2012; Chen et al., 2013; Resendiz et al., 2013).

### Conflict of interest statement

The authors declare that the research was conducted in the absence of any commercial or financial relationships that could be construed as a potential conflict of interest.
